# Sensory Recovery After Free Muscle Flap Reconstruction—A Clinical Study of Protective and Discriminative Function of Free Gracilis and Latissimus Dorsi Muscle Flaps Without Neurotization

**DOI:** 10.3390/medsci13040262

**Published:** 2025-11-07

**Authors:** Maximilian C. Stumpfe, Moritz Billner, Marc Hellweg, Maximilian Hirschmann, Rakan R. Al-Turki, Celena A. Sörgel, Vadym Burchak, Nikolaus Wachtel, Denis Ehrl

**Affiliations:** 1 Department of Plastic, Reconstructive and Hand Surgery, Burn Centre for Severe Burn Injuries, Nuremberg Clinics, University Hospital, Paracelsus Medical University, Breslauerstrasse 201, 90471 Nuremberg, Germany; 2 Division of Hand, Plastic and Aesthetic Surgery, University Hospital, LMU Munich, Marchioninistraße 15, 81377 Munich, Germany

**Keywords:** free muscle flap, sensory recovery, gracilis flap, latissimus dorsi flap, nerve coaptation

## Abstract

Background/Objectives: Free gracilis (GM) and latissimus dorsi muscle (LDM) flaps are reliable options for complex defect coverage, but long-term sensory outcomes remain underexplored. Sensory impairment, especially the loss of protective cutaneous sensation, increases the risk of injury, thermal damage, and ulceration in reconstructed areas. This study aimed to systematically assess multidimensional sensory recovery after free muscle flap (FMF) reconstruction. Methods: In a prospective single-center study, 94 patients (49 GM, 45 LDM) underwent standardized sensory testing following FMF transfer. Five modalities were evaluated: pressure detection (Semmes-Weinstein monofilaments), vibration perception, two-point discrimination (2PD), sharp–dull differentiation, and temperature differentiation. Measurements were compared to contralateral healthy skin (CHS). Subgroup analyses were performed by anatomical region (head, trunk, extremities). Results: All sensory modalities were significantly impaired in FMF compared to CHS (*p* < 0.0001). Mean pressure thresholds were markedly higher in FMF (248.8 g) versus CHS (46.8 g). Vibration perception scores were reduced (FMF 3.97 vs. CHS 5.31), and 2PD was significantly poorer (11.6 cm vs. 4.7 cm). Sharp–dull and thermal discrimination were largely absent in FMF (positivity rates < 20%), with 58.5% of patients demonstrating only deep pressure sensation (≥300 g). No significant differences were found between GM and LDM in most modalities, except for worse 2PD in GM. Subgroup analyses confirmed uniform deficits across all anatomical regions. Conclusions: FMFs without neurotization result in profound, persistent sensory deficits, particularly the loss of protective sensation. Clinically, fascio-cutaneous flaps with nerve coaptation should be considered in functionally critical regions. Future strategies should focus on neurotization techniques to enhance sensory recovery.

## 1. Introduction

Since its inception around 1600 BC, the restoration of form and function using muscle flap surgery has been a cornerstone of reconstructive surgery [1]. In addition to its main objectives of providing soft tissue coverage and functional reconstruction after trauma, tumor resection, or chronic wound complications, research on the sensory properties of transplanted tissue is becoming increasingly important [2]. The free gracilis muscle (GM) flap and the latissimus dorsi muscle (LDM) flap are widely used due to their anatomical reliability, versatility, and acceptable donor site morbidity [3,4].

Although primary surgical objectives focus on achieving durable coverage and functional integration, the long-term sensory outcomes of free muscle flaps (FMF) are critical yet underinvestigated. Sensory impairment, particularly loss of protective cutaneous sensation, increases the risk of injury, thermal burns, and pressure-related complications in areas reconstructed by free flaps. This negatively impacts postoperative quality of life [2,5]. Reduced sensory feedback negatively affects subjective quality of life as well, as the affected areas are often perceived as foreign bodies [2].

Current research indicates that muscle flap procedures without targeted sensory nerve coaptation result in significant postoperative restriction of all sensory modalities [6,7,8,9,10,11,12,13,14,15,16,17,18,19].

Although the existing literature is informative in terms of sensory reductions following free flap surgery, it is predominantly limited by a small number of studies, by small patient cohorts, or by a focus on isolated sensory modalities. Consequently, there is a lack of comprehensive data on multidimensional sensory recovery.

This study addressed this issue by systematically assessing the long-term sensory performance of FMF in five modalities—pressure detection, vibration perception, two-point discrimination, sharp-blunt discrimination, and temperature differentiation—in a large, clinically diverse cohort using standardized, validated protocols. Comparative evaluation between the flap and contralateral healthy skin served as controls.

## 2. Materials and Methods

### 2.1. Study Population and Surgical Technique

In a single-center study, we compared the sensory development of free muscle flaps, with a specific focus on free gracilis and latissimus dorsi flaps. The study took place at a Level 1 hospital in Germany from May 2019 to April 2021. It was a single-surgeon study, with one experienced senior physician performing all free flaps.

Patients scheduled to undergo reconstructive procedures involving free microvascular gracilis or latissimus dorsi muscle flaps due to infection, trauma, or malignancy were included in the study. We followed a standardized surgical protocol for flap harvesting and microvascular anastomosis. Ischemia time was documented in each case, and split-thickness skin grafts (STSGs) were used to cover the muscle flap at the recipient site. After wound healing, patients were instructed to wear compression garments continuously for at least six months.

The inclusion criteria were all patients receiving a free gracilis or latissimus dorsi muscle flap and an age over 18 years, capacity to consent, and a follow-up of at least three months for the study group (see Table 1). Cases involving urgent flap revisions due to complications such as arterial or venous thrombosis, hematoma, or flap loss exceeding 10% were excluded. Additional exclusion criteria included patients who were lost to follow-up.

Written informed consent was obtained from each participant. The study was approved by the institutional research ethics committee (reference numbers 17-630 and 21-0475, Approval date: 2021-07-16) and conducted according to the 1964 Helsinki Declaration and subsequent amendments or comparable ethical standards.

### 2.2. Sensory Evaluation

**Figure 1:**
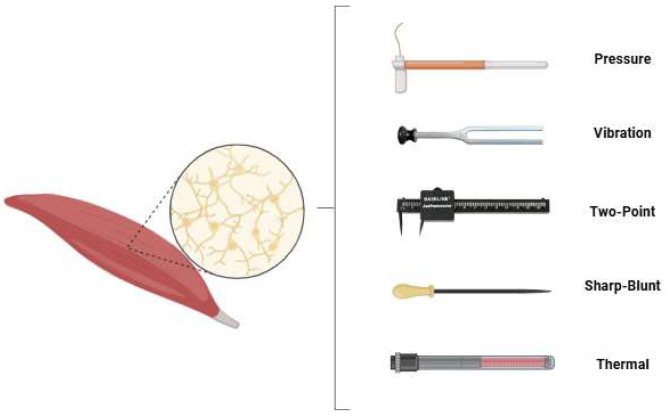
The sensory testing modalities applied in this study were touch-pressure threshold, vibration sensation, two-point discrimination, sharp–dull differentiation and temperature differentiation.

In all participating patients, sensory testing was performed by the same examiner for free gracilis and latissimus dorsi flaps. The examination included five domains: touch-pressure threshold, vibration sensation, two-point discrimination, sharp–dull discrimination, and temperature differentiation (see Figure 1).

#### 2.2.1. Touch-Pressure Threshold (Monofilaments)

Pressure detection thresholds were assessed using calibrated Semmes-Weinstein monofilaments (Baseline 12-1662 Tactile Monofilament, Fabrication Enterprises Inc., White Plains, NY, USA).

The test battery was performed according to a standardized protocol. On this scale, lower gram values reflect better sensory function, with smaller values indicating greater sensitivity to light touch. No sensory perception occurred at a force of 500 g, while a threshold of 300 g indicated minimal sensitivity.

#### 2.2.2. Vibration Sensation

The vibratory sensation was evaluated on the basis of a predetermined study protocol using a DocCheck “Plong”) tuning fork (DocCheck Tools, Cologne, Germany) with an eighth-marked scale, Vibration sense was measured on a scale where higher values indicated better sensory perception. A score of 1 represented poor vibration sense, and a score of 8 represented excellent vibration sensitivity.

#### 2.2.3. Two-Point Discrimination

Two-point discrimination was measured using a Baseline^®^ Aesthesiometer (Fabrication Enterprises Inc., White Plains, NY, USA). The smallest distance that could be reliably distinguished was recorded. Two-point discrimination (2PD) was assessed in centimeters, with lower values indicating better discriminatory ability. A distance of 14 mm reflected very poor discrimination capacity, and a value of 15 mm indicated an absence of sensation.

#### 2.2.4. Sharp–Dull Discrimination

We assessed the ability to discriminate between sharp and dull stimuli qualitatively using a 6.65 monofilament (sharp stimulus) and a brass ball embedded in a multifunction tuning fork (blunt stimulus). A positive response was defined as the correct identification of the stimulus as sharp or blunt in at least three out of five trials, a negative test vice versa.

#### 2.2.5. Temperature Differentiation

The thermal sensation was evaluated using a multifunction tuning fork (DocCheck “Plong”; DocCheck Tools, Cologne, Germany). The plastic base of the tuning fork, warmed by the examiner’s hand, served as the warm stimulus. The unheated metal portion represented the cold stimulus. A result was considered valid if at least three out of five responses correctly identified the stimulus.

### 2.3. Subgroup Analysis

Subgroup comparisons were conducted separately for pressure sensitivity, vibration perception, and two-point discrimination for the following anatomical regions: the head, extremities, and trunk. Specifically, the free muscle flap (FMF) was compared against the untreated, anatomically correlating healthy skin (CHS) in each anatomical location. Furthermore, intra-group comparisons were performed within the FMF cohort to assess region-specific differences. This subgroup structure enabled regional differences within the FMF subgroup and differences between FMF tissue and healthy controls in anatomically matched regions to be systematically evaluated. A detailed list of the subgroup analyses performed is provided in Table 2.

### 2.4. Statistical Analysis

We analyzed patient demographics and performed a subgroup analysis using descriptive statistics. Descriptive statistics are expressed as the mean ± standard deviation (SD) for parametric data and the median ± SD for nonparametric data. We used a paired sample *t*-test or Wilcoxon’s signed-rank test for significance tests. Significance was set at a *p*-value of less than 0.05. All analyses were performed using GraphPad Prism Version 9 (GraphPad Software Inc., La Jolla, CA, USA).

## 3. Results

### 3.1. Total Patient Collective Demographics

A total of 94 patients (44 male and 50 female) were included in this study. The average age at the time of examination was 54.1 years (range: 16–93 years). The mean follow-up time was 16.9 months (range 3.0–52.9 months, SD +/− 13.4).

### 3.2. Flap Characteristics

In total, 94 soft tissue defects were reconstructed using free muscle transfers (49 GM and 45 LDM). The underlying etiologies included tumor resections in 33 cases (35.1%), chronic non-healing wounds in 28 cases (29.8%), and post-traumatic defects in 33 cases (35.1%).

**Figure 2:**
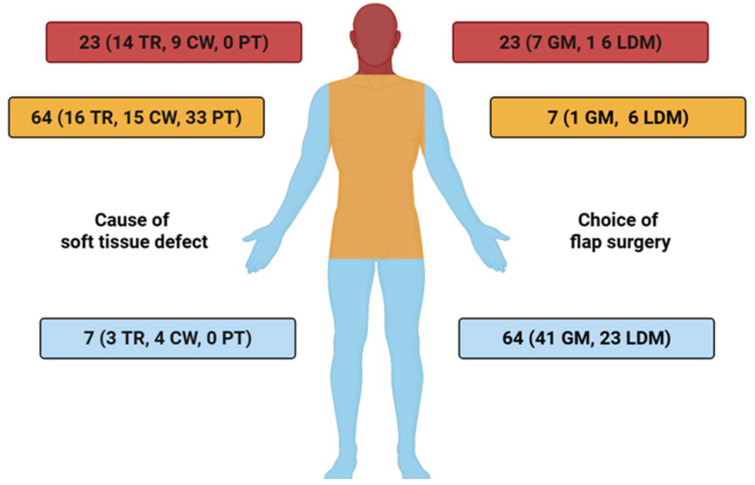
Anatomical distribution of free muscle flap reconstructions (GM = Gracilis muscle, LDM = Latissimus dorsi muscle), stratified by defect etiology: tumor resection (TR), chronic wounds (CW), and post-traumatic injuries (PT) TR = tumor resection, CW = chronic wound, PT = post-traumatic.

A detailed overview of the distribution of free muscle flaps across anatomical recipient sites, stratified by defect etiology, is presented in Figure 2.

### 3.3. Sensory Evaluation

#### 3.3.1. Pressure Sensitivity (Monofilaments)

**Figure 3:**
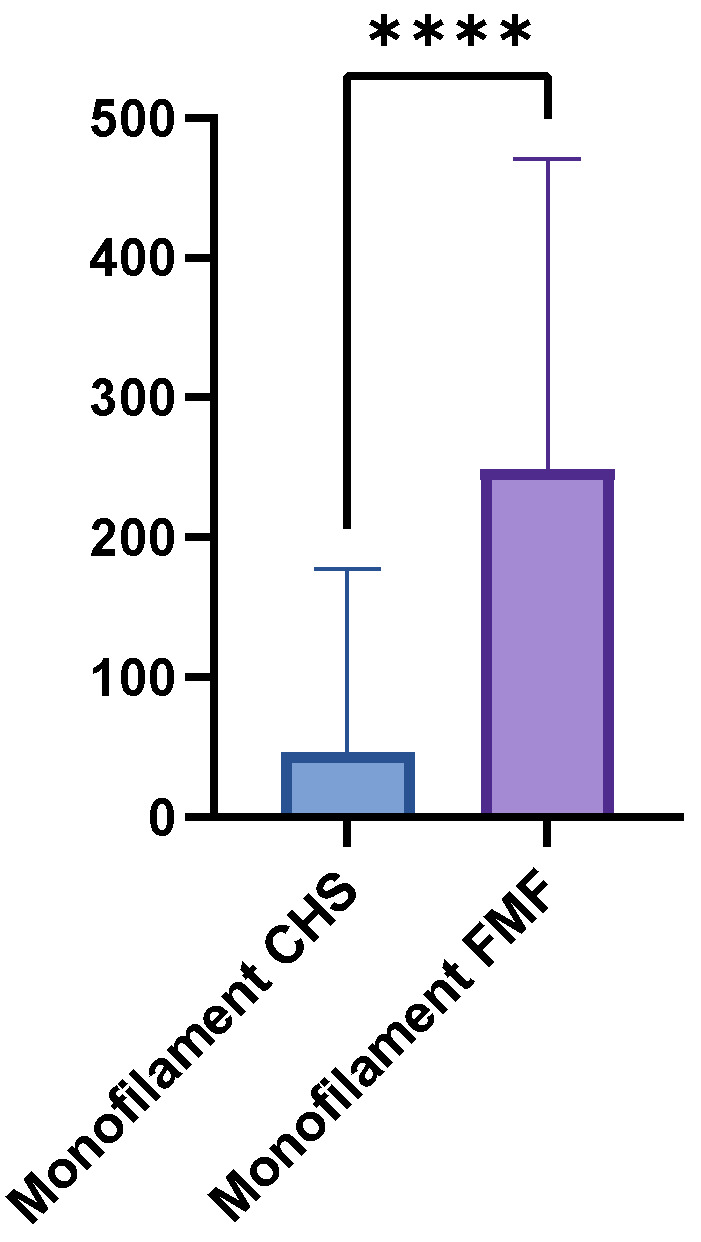
Boxplot comparing Semmes-Weinstein monofilament thresholds in FMF tissue versus CHS. Higher thresholds indicate reduced pressure sensitivity (*p* < 0.0001). **** = extremely significant.

The mean monofilament threshold was higher in the FMF group (248.8 g, SD = 221.7, range = 0.07–500 g) than in the CHS group (46.8 g, SD = 130.6, range = 0–500 g). The mean difference between the two groups was 202 g, which a paired *t*-test confirmed to be statistically highly significant (*p* < 0.0001) (Figure 3). Between the GM flaps and the LDM flaps were no significant differences (*p* = 0.1043). Table 3 provides a detailed overview of the monofilament results.

#### 3.3.2. Vibration Sensation

**Figure 4:**
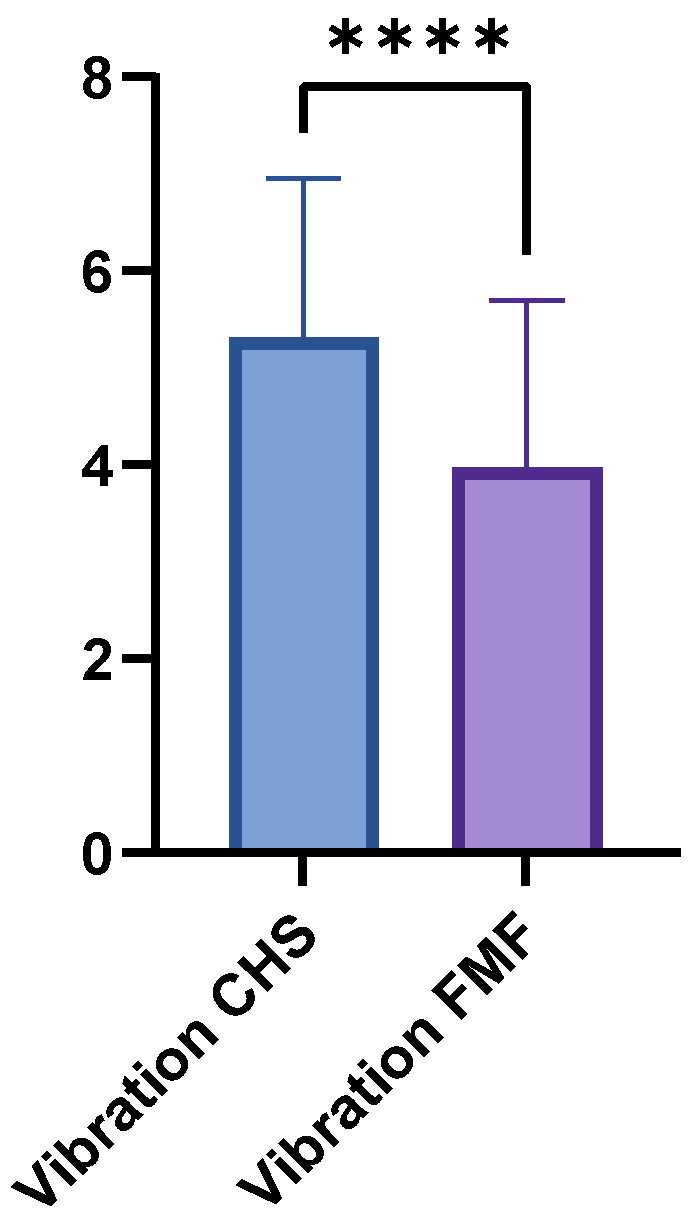
Comparison of vibration sensation scores between FMF and CHS tissues. FMF scores were significantly reduced across all regions (*p* < 0.0001). **** = extremely significant.

The FMF group achieved a mean vibration score of 3.97 (±1.73; range 0–7), whereas the CHS group achieved a higher mean score of 5.31 (±1.65; range 1–8; *p* < 0.0001) (Figure 4). LDM and GM flaps showed no significant differences in vibration sensation (*p* = 0.1364).

#### 3.3.3. Two-Point Discrimination (2PD)

**Figure 5:**
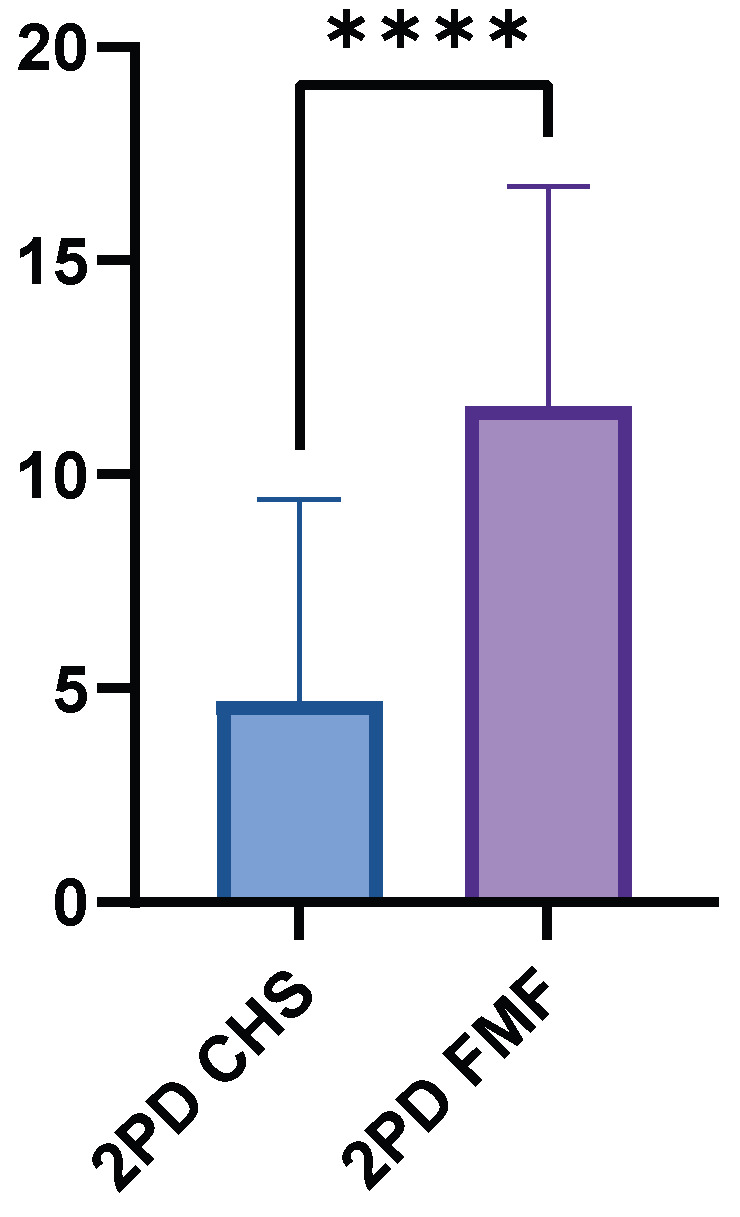
Two-point discrimination performance in FMF compared to healthy control skin. FMF regions demonstrated markedly increased threshold distances (*p* < 0.0001). **** = extremely significant.

The FMF group had a higher mean 2PD value of 11.60 cm (±5.15, range 0.8–15.0 cm), while the CHS group had a significantly lower mean value of 4.71 cm (±4.70, range 0.5–15.0 cm). The mean difference between the two groups was 6.88 cm (*p* < 0.0001) (Figure 5). The GM flaps showed significantly higher 2PD than the LDM flaps (*p* < 0.0001).

#### 3.3.4. Sharp–Dull Discrimination

In the sharp–dull discrimination test, significant differences were observed between FMF and CHS findings. In total, 74 patients showed a negative result in FMF while being classified as positive in CHS, whereas only 2 patients showed a positive result in FMF with a negative result in CHS. Application of the McNemar test confirmed this difference to be highly significant (*p* < 0.001). A detailed overview of the results is presented in Table 4. A separate analysis of the GM and LDM flaps also showed a significant difference in the McNemar test (*p* < 0.001).

#### 3.3.5. Thermal Discrimination

A clear disparity in thermal sensation outcomes was identified between FMF and CHS. Overall, 82 cases were classified as positive in the contralateral healthy skin (CHS) group but negative in the FMF group, whereas only one case showed the opposite pattern. This led to a statistically significant finding when the McNemar test was used (*p* < 0.001). A comprehensive summary of the results is provided in Table 5. These results were also confirmed in a separate analysis of the GM and LDM flaps.

### 3.4. Subgroup Analyze

#### 3.4.1. Pressure Sensitivity (Monofilaments)

Comparative subgroup analysis revealed significant differences between all FMF groups and their contralateral healthy skin (CHS) Additional significant differences were observed between Head FMF and Trunk FMF (*p* = 0.0480), and between Extremity FMF and Trunk FMF (*p* = 0.0494). All detailed *p*-values are presented in Table 6.

#### 3.4.2. Vibration Sensation

Subgroup comparisons revealed highly significant differences between Head FMF and Head CHS, and between Extremity FMF and Extremity CHS. Further relevant differences were observed between Head FMF and Trunk FMF, and between Extremity FMF and Trunk FMF. Full statistical details are provided in Table 7.

#### 3.4.3. Two-Point Discrimination

A statistical difference was identified between Head FMF and Head CHS, as well as between Extremity FMF and Extremity CHS. In contrast, comparisons involving Trunk FMF generally showed no significant differences, including the comparison with Trunk CHS. A full overview of all pairwise analyses can be found in Table 8.

## 4. Discussion

This prospective, single-surgeon clinical study observed significant long-term sensory impairment following free muscle flap surgery. The results show that non-neurotized muscle grafts lead to pronounced sensory deficits in the long term. In all five modalities examined—pressure perception, vibration perception, static two-point discrimination, sharp-blunt differentiation, and thermal perception—sensitivity in the graft was significantly reduced compared to the contralateral healthy skin. It is especially relevant that 58.5% of patients only had deep pressure perception in the sense of thresholds ≥300 g, while finer qualities such as vibration and temperature differentiation were absent in the vast majority of cases.

The present results support and supplement the existing findings in the published literature. Engelhardt et al. already described significant limitations in epikritical sensitivity after free gracilis transplantation despite low lifting morbidity [20]. In a comparison of gracilis, latissimus dorsi, and ALT flaps, Rothenberger et al. demonstrated that although the gracilis leaves the smallest anesthetic area, the overall return of sensitivity remains insufficient, while the ALT flaps showed the best results [7]. Tremp et al. reported a partial return of two-point discrimination, while pressure, vibration, and thermal sensitivity were hardly recovered [8]. This finding is almost identical to the results of the present study. Huber and Kim observed reinnervation from the periphery to the center in larger groups and also documented a significant restriction of all modalities [21,22]. Puonti and Lähteenmäki were unable to demonstrate complete recovery of sensitivity even after two years following TRAM or ms-TRAM transplants [15,22]. Studies on LDM flaps show that although deep pressure perception is often preserved, fine pressure and thermal sensitivity are practically impossible to regain [9,10,11,14,16,19]. Gordon et al. confirmed in a review that complete sensory reinnervation is not to be expected [23]. Overall, all studies paint a consistent picture: non-neurotized muscle grafts do not develop sufficient protective sensitivity, meaning that functional limitations remain permanent.

The decision to choose between muscle and fascio-cutaneous flaps requires careful consideration in terms of the available results. Free muscle flaps such as the gracilis or latissimus dorsi are impressive due to their anatomical reliability, low morbidity, and versatility [3,4,24]. The major advantage of muscle flap flaps is the dimensions that can be achieved. All sizes of soft tissue defects can be adequately reconstructed. The latissimus dorsi flap can be used for large defects and the free gracilis flap for small to medium defects [4,25]. The size of the latissimus dorsi flap makes it possible to completely cover the sole of the foot [25,26]. It should also be noted that muscle flaps show an excellent tendency to atrophy and thus adapt to their surroundings, resulting in good adhesion to the underlying tissue [25]. In the case of fascio-cutaneous free flaps, which tend to be bulky, shear forces cause a feeling of instability in the absence of adhesion [24,26]. Ultimately, the free muscle flaps also regain their deep sensitivity and are therefore not completely insensitive. However, they show significant deficits in terms of sensitivity. The available data confirm that without nerve capacitation, neither protective nor differentiated sensitivity is regained, which significantly increases the risk of injury and ulceration [27,28]. These results are consistent with earlier studies that also described pronounced deficits in free muscle grafts [8,9,10,11,12,14,16,19,20,21,22]. Coaptation of a sensory receptor nerve to the motor nerves of the flap has been described in studies as a potential strategy for optimizing sensory recovery. This technique has been investigated in smaller series, for example by Potparic et al., who showed in a series of 22 flaps (12 muscle flaps and 10 fascio-cutaneous flaps) that motor-sensitive capacitation or onlay nerve grafts enable a certain degree of reinnervation, which remains limited in terms of quality [29]. In the available studies on LDM and GM flaps, it was found that deep pressure sensitivity is preserved even without coaptation, while fine sensitivity rarely returns [9,10,14]. Gordon et al. also note that the quality of motor-sensory coaptation is not comparable to that of direct sensory nerve coaptation [23]. Significantly better results are reported for fascio-cutaneous flaps with sensory nerve capacity. Santanelli demonstrated rapid reinnervation within six months [30]. Small fascio-cutaneous flaps reinnervated by nerve coaptation showed the fastest and most complete reinnervation [29]. In functionally highly relevant regions, such as the sole of the foot or the hand, a fascio-cutaneous flap with sensory nerve capacity should be considered [24]. In situations where volume reconstruction, reliable defect coverage, or functional aspects are paramount and sensitivity plays a secondary role, muscle flaps remain the indicated treatment [7,15,23,31,32,33].

One limitation of this study is that sensory assessment was performed only in the central zone of the flap. Since previous studies suggest that reinnervation occurs from the periphery toward the center, the degree of peripheral sensory recovery in this cohort may have been underestimated. The absence of preoperative baseline data in cases involving chronic wounds or post-traumatic defects may have distorted some sensory results, as preexisting neuropathy could not be entirely ruled out. Another limitation is the heterogeneity of the cohort regarding both the underlying cause of tissue loss and the type of flap used. This variability may have introduced bias, although subgroup analyses demonstrated consistent results across etiologies and anatomical regions, supporting the robustness of the findings.

The study has important strengths: with 94 patients it represents one of the largest prospective cohorts on sensory recovery after free muscle flap transfer, all operations were performed by a single experienced surgeon using standardized techniques, and sensory evaluation was conducted with validated multimodal testing protocols across five modalities, ensuring a high level of methodological rigor and reliability of the findings.

## 5. Conclusions

In conclusion, our study shows that free gracilis and latissimus dorsi muscle grafts without neurotization exhibit pronounced sensory deficits in the long term, resulting in a significant risk of injury, thermal damage, and ulceration.

Clinically, where functional sensitivity is essential—such as on the sole of the foot, the hand, or other highly stressed regions—a fascio-cutaneous flap with nerve coaptation is preferable to a muscle graft. Muscle flaps remain relevant in situations where volume reconstruction, secure defect coverage, or functional aspects of muscle activity are paramount and sensitivity is of secondary importance.

In addition, patients should be given comprehensive preoperative information about the permanent loss of sensitivity and the associated risks. From a surgical point of view, it is necessary to further evaluate strategies such as targeted nerve coaptation or innovative neurotization procedures in order to improve the sensory outcomes of free muscle grafts in the future.

## Figures and Tables

**Table 1:** Overview of the inclusion and exclusion criteria for the study.

Inclusion Criteria	Exclusion Criteria
Free gracilis oder latissimus dorsi muscle flap	Urgent flap revision due to complications
Age over 18 years	Flap loss
Capacity to consent	Loss of follow up
Follow-Up of at least three months	

**Table 2:** Subgroup comparisons performed between free muscle flaps (FMF) and contralateral healthy skin (CHS) by anatomical region, including intragroup comparisons within the FMF cohort.

Comparison	Group A	Group B
Head FMF vs. CHS	FMF—Head region	CHS—Head region
Extremity FMF vs. CHS	FMF—Extremity	CHS—Extremity
Trunk FMF vs. CHS	FMF—Trunk	CHS—Trunk
Head FMF vs. Trunk FMF	FMF—Head region	FMF—Trunk
Head FMF vs. Extremity FMF	FMF—Head region	FMF—Extremity
Trunk FMF vs. Extremity FMF	FMF—Trunk	FMF—Extremity

**Table 3:** Distribution of pressure sensitivity according to Semmes-Weinstein monofilament force thresholds in the FMF group versus contralateral healthy skin (CHS), including clinical interpretation by sensory level.

Force (g)	Total FMF *(n)*	Total CHSH *(n)*	Interpretation
0.07 g	4	21	Normal sensation
0.4 g	7	45	Diminished light touch
2 g	15	9	Diminished protective sensation
4 g	13	6	Loss of protective sensation
300 g	21	7	Deep pressure sensation only
500 g	34	6	No sensation

**Table 4:** Cross-tabulation of sharp–dull discrimination outcomes in FMF versus contralateral healthy skin. Significant asymmetry in recovery (McNemar test, *p* < 0.0001).

	Negativ CHS	Positiv CHS	Total	*p*-Value
Negativ FMF	2	74	76	<0.0001
Positiv FMF	2	16	18	
Total	4	90	**94**	

**Table 5:** Thermal discrimination test results comparing FMF with contralateral healthy skin, marked sensory impairment in the FMF cohort (McNemar test, *p* < 0.0001).

	Negativ CHS	Positiv CHS	Total	*p*-Value
Negativ FMF	3	82	85	<0.0001
Positiv FMF	1	8	9	
Total	4	90	94	

**Table 6:** Subgroup analysis for pressure sensitivity (monofilaments): inter- and intragroup comparisons among FMF and CHS by anatomical site. Statistically significant differences are highlighted; n.s. = not significant. * = significant; ** = very significant.

	Head FMF	Head CHS	Extr. FMF	Extr. CHS	Trunk FMF	Trunk CHS
Head FMF		** (0.0018)	n.s. (0.9955)		* (0.0480)	
Head CHS	** (0.0018)					
Extr. FMF	n.s. (0.9955)			** (0.0026)	* (0.0494)	
Extr. CHS			** (0.0026)			
Trunk FMF	* (0.0480)		* (0.0494)			* (0.0156)
Trunk CHS					* (0.0156)	

**Table 7:** Subgroup analysis for vibration sensation: comparisons among FMF and CHS by anatomical site. All values are *p*-values from paired *t*-tests or Wilcoxon rank-sum tests. * = significant; ** = very significant; *** = extremely significant.

	Head FMF	Head CHS	Extr. FMF	Extr. CHS	Trunk FMF	Trunk CHS
Head FMF		*** (<0.0001)	* (0.0435)		** (0.0076)	
Head CHS	*** (<0.0001)					
Extr. FMF	* (0.0435)			*** (<0.0001)	* (0.0331)	
Extr. CHS			*** (<0.0001)			
Trunk FMF	** (0.0076)		* (0.0331)			* (0.0469)
Trunk CHS					* (0.0469)	

**Table 8:** Subgroup analysis for static two-point discrimination: comparisons among FMF and CHS groups by anatomical site. No significant difference was found in trunk-related comparisons. *** = extremely significant.

	Head FMF	Head CHS	Extr. FMF	Extr. CHS	Trunk FMF	Trunk CHS
Head FMF		*** (<0.0001)	n.s. (0.2947)		n.s. (0.7542)	
Head CHS	*** (<0.0001)					
Extr. FMF	n.s. (0.2947)			*** (<0.0001)	n.s. (0.5928)	
Extr. CHS			*** (<0.0001)			
Trunk FMF	n.s. (0.7542)		n.s. (0.5928)			n.s. (0.0625)
Trunk CHS					n.s. (0.0625)	

## Data Availability

The datasets generated and analyzed in the current study are not publicly available due to data protection regulations. Access to data is limited to the researchers who have obtained permission for data processing. Further inquiries can be made to the corresponding author.

## Abbreviations

The following abbreviations are used in this manuscript:

GM	Gracilis muscle
LDM	Latissimus dorsi muscle
FMF	Free muscle flap
STSG	Split-thickness skin graft
2PD	Two-point discrimination
CHS	Correlating healthy skin
SD	Standard deviation
TR	Tumor resection
PT	Post-traumatic
CW	Chronic wound
ALT	Anterior lateral thigh
